# Pectoralis Major Tendon Rupture in an Occupational Medicine Setting: A Case Report

**DOI:** 10.7759/cureus.55569

**Published:** 2024-03-05

**Authors:** Rasha Saeed, Scott E Hardy, Alya Khan

**Affiliations:** 1 Occupational Medicine, University of California, Irvine, Irvine, USA; 2 Medicine/Occupational Medicine/Medical Toxicology, University of California, Irvine Medical Center, Irvine, USA; 3 Medicine/Occupational and Environmental Medicine, University of California, Irvine, Irvine, USA

**Keywords:** non-cardiac chest pain, work-related injury, occupational injuries, pectoralis major rupture, pectoralis majors injury

## Abstract

Pectoralis major (PM) rupture is a rare injury, commonly misdiagnosed, that affects mostly young male athletes aged 20-40 years. This type of injury is typically associated with weight lifting, especially bench pressing. In an occupational medicine setting, it is extremely rare and not much reported in the literature. We present the case of a 30-year-old trauma technician male who presented with right shoulder and chest pain following a popping sensation while pushing in full momentum a patient on a gurney accidentally set on break mode. PM rupture was suspected clinically. Magnetic resonance imaging confirmed the diagnosis and revealed a complete rupture of the sternal head of PM. Surgical reconstruction was performed to restore the anatomy and functionality of the shoulder girdle.

## Introduction

The pectoralis major (PM), a fan-shaped multi-pennate muscle, is the largest muscle of the anterior chest and has a clavicular head originating from the medial clavicle and a sternocostal head originating from the sternum. Both heads are inserted at the proximal humerus. The primary functions are flexion, adduction, and internal rotation of the humerus [1]. Injuries of this muscle are rare and occur in individuals involved in recreational and physical activities [2]. A high degree of clinical suspicion is needed to avoid a misdiagnosis of this rare injury. We present the case of a 30-year-old trauma technician at a level-one trauma center who suffered an injury while working.

## Case presentation

A 30-year-old right-hand dominant male, working as a trauma technician in a level I trauma center, presented with sudden right shoulder and chest pain and a “pop” sensation after pushing with extended arms in full momentum a patient weighing around 90 kg on a gurney with the brake engaged. The pain was described as a sharp, tearing sensation, rated 6/10 when the injury occurred. He denied a history of smoking, or being on any medication including anabolic steroids, and had no family history of a similar injury.

**Figure 1:**
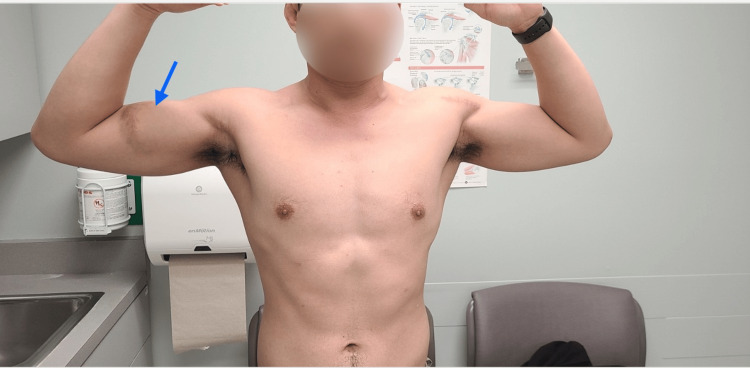
Preoperative physical examination is remarkable for right upper limb ecchymosis and the right chest edema, secondary to the ruptured pectoralis major.

On physical examination, a right anterior-medial upper arm bruise was noticed, as well as right upper chest wall deformity with loss of lateral margin of the PM muscle, with dropped nipple sign associated with significant pain on resisted arm adduction and modest pain with passive arm abduction. Weakness in adduction and internal rotation of the shoulder was also noted and recorded. Neurovascular examination was intact (Figure 1).

**Figure 2:**
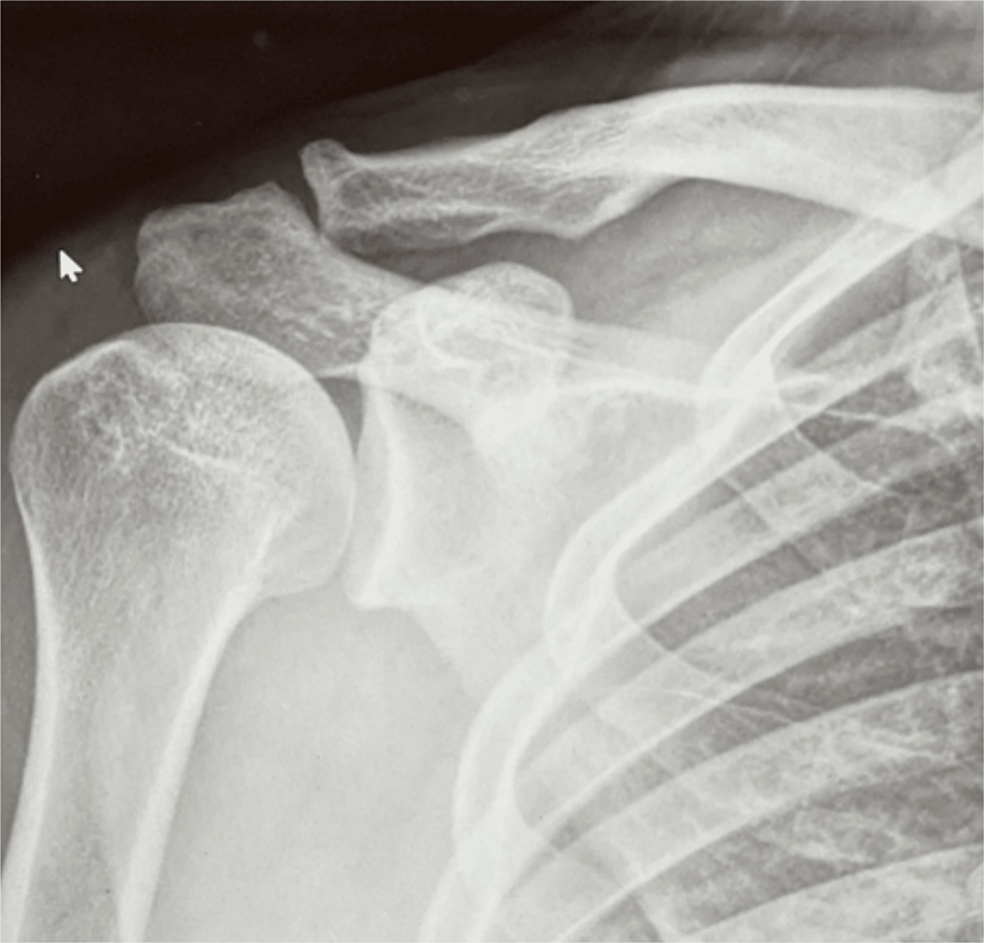
Right shoulder X-ray. No fracture or dislocation is noticed.

An X-ray of the right shoulder was performed to rule out proximal humerus fracture and shoulder dislocation, which was found to be normal (Figure 2).

**Figure 3:**
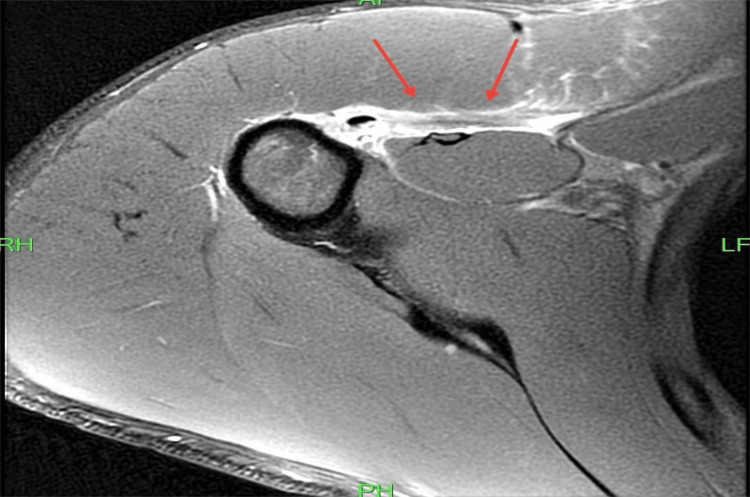
Right shoulder magnetic resonance imaging. Pectoralis major sternal head tendon rupture with fluid collection (red arrow). RH: right head; LF: left foot; PH: posterior head

An urgent magnetic resonance imaging (MRI) scan without contrast revealed the full thickness of sternal PM rupture. PM muscle retraction was also evident. No biceps tendon or rotator cuff tear was noted, and no fracture was noticed (Figure 3).

**Figure 4:**
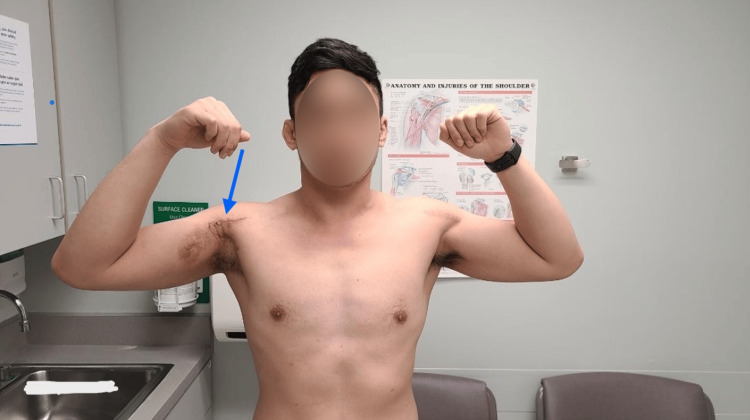
Right incision scar eight weeks postoperatively (blue arrow).

Surgical repair of the PM tendon was performed. Taking into consideration that this heavy laborer needed to return to his occupation, surgical management was performed at nine weeks post-injury. Intraoperatively, a retracted torn sternal head was noted, approximately 5 cm away from the anatomic attachment. Postoperatively, the shoulder was stabilized with a sling for four weeks, followed by physical therapy rehabilitation in two phases, passive range of motion for six weeks, followed by active muscle strengthening for six weeks (Figure 4).

## Discussion

PM rupture is a rare injury that was first described by Patisser in 1822 [3]. It is more common in males 20-40 years of age. It occurs mainly in sports such as weight lifting, specifically in bench pressing, contact sports, and gymnastics [4]. Altercation and seatbelt blunt trauma have also been reported [5]. PM rupture in occupational settings is less common and can occur due to heavy labor, falls, and direct trauma. Anabolic steroids and levofloxacin fluoroquinolone antibiotics increase the risk of PM tendon rupture [6,7].

Anatomy/physiology

PM muscle is the largest muscle of the anterior chest. It is fan-shaped and consists of two segments, clavicular and sternal heads, that join together and attach to the intertubercular sulcus of the humerus. It is innervated by lateral pectoral (C5-C7) and medial pectoral nerves (C8-T1), which stem from the brachial plexus. PM function is flexion, adduction, and internal rotation of the arm. It causes flexion of the extended arm (clavicular head) and extension of the flexed arm (sternal head) [8]. In most cases, PM rupture occurs when a force is applied to the PM muscle while in eccentric contraction.

Differential diagnosis

Proximal humerus fracture, shoulder dislocation, biceps long tendon tear, rotator cuff tendon tear, and medial pectoral nerve entrapment need to be considered in similar presentations of PM injury [9].

Imaging

X-ray has a limited role in detecting PM injury. It helps in detecting bone avulsion and shoulder dislocation. Ultrasound has proven to be an effective and relatively inexpensive way to identify and even locate a PM rupture. It has been recommended as a means of avoiding delay in surgery when PM rupture is suspected. MRI, on the other hand, is the modality of choice in most cases of suspected PM tendon rupture. It also helps in the treatment of choice when the tear is partial versus complete and acute versus chronic. T2-weighted axial imaging seems to be most effective, especially in the acute and subacute settings, with T1-weighted imaging helpful in the case of chronic rupture [10].

Treatment

Surgical repair is preferred to restore function in acute injuries and in patients with highly physically demanding jobs in addition to cosmetic purposes. Non-surgical treatment, which includes physical therapy and pain management, can be reserved for elderly, low-demand, and less active patients with medical comorbidities, as well as patient preferences [11]. Surgical treatment, preferably performed within the first eight weeks after the injury, has a significantly better outcome than conservative treatment or delayed repair [12,13].

## Conclusions

PM rupture is an uncommon sport-related injury and a rare work-related injury encountered in occupational medicine practice. Regardless of the cause, early recognition and surgical anatomic repair yield the best outcomes. Surgical repair within eight weeks from the injury followed by physical therapy provides optimal outcomes for workers with PM rupture to return to their pre-injury occupational activities.
